# Nanosensors based on polymer vesicles and planar membranes: a short review

**DOI:** 10.1186/s12951-018-0393-7

**Published:** 2018-08-30

**Authors:** Mohamed El Idrissi, Claire Elsa Meyer, Luisa Zartner, Wolfgang Meier

**Affiliations:** Department of Chemistry, University of Basel, Mattenstrasse 24a, 4002 Basel, Switzerland

**Keywords:** Nanosensor, Sensor, Vesicle, Polymer, Polymersome, Planar membrane

## Abstract

This review aims to summarize the advance in the field of nanosensors based on two particular materials: polymer vesicles (polymersomes) and polymer planar membranes. These two types of polymer-based structural arrangements have been shown to be efficient in the production of sensors as their features allow to adapt to different environment but also to increase the sensitivity and the selectivity of the sensing device. Polymersomes and planar polymer membranes offer a platform of choice for a wide range of chemical functionalization and characteristic structural organization which allows a convenient usage in numerous sensing applications. These materials appear as great candidates for such nanosensors considering the broad variety of polymers. They also enable the confection of robust nanosized architectures providing interesting properties for numerous applications in many domains ranging from pollution to drug monitoring. This report gives an overview of these different sensing strategies whether the nanosensors aim to detect chemicals, biological or physical signals.

## Background

Polymer sensors play an important role in monitoring our environment and could become soon an essential part of our modern sensor devices [1, 2]. Among sensor devices, polymers are the most commonly used materials and do not cease to be the topic of intensive investigations [3]. Two particular polymer materials that can have significant impact on nanosensors devices are worth to focus on. The first class of polymer is represented by the polymersomes or artificial polymer vesicles based on the self-assembly of polymers. Their characteristic structure allows the encapsulation of components as well as the functionalisation of the vesicle membrane [4]. The main advantage of these versatile nanocapsules lies in their great tunability accessible through the structure of the polymers [5]. Planar polymer membranes constitute the second class of polymer material, the latter can be formed from different type of polymers nanoporous, conducting or block-copolymer for example. They represent an excellent alternative to study biological membrane without to have to deal with the complexity of biological structures [6]. These two types of polymer material have been studied for the construction of nanosensors. Herein, the term nanosensor is discussed in a broad sense, and refer to any sensing process occurring at the nanoscale, either it is because of a nanomembrane, a nanopore or a nano-vesicle, to name few examples. This review intends to provide a short updated and non-exhaustive overview on those polymer-based nanosensors. We will mainly focus on the different kind of polymersomes and planar polymer membranes as well as their characteristics and functionalities through selected studies from literature.

## Polymer vesicles for nanosensors

### Introduction

Polymersomes are interesting artificial vesicles to be considered for nanosensors due to their strong responsiveness to variety of stimuli. For example, the sensing mechanism of the polymersomes based on detectable fluorescence or color transition can be caused by diverse environmental triggers such as pH and redox potential. In recent years, nanosized polymersomes have also been developed to detect different ions, small molecules, macro-molecules or enzymes. In comparison to polymer micelles or nanoparticles, polymer vesicles are able to carry not only hydrophobic but also hydrophilic cargo within their core. In addition, many polymersomes are nontoxic for organisms but exhibit thicker and more robust membranes compared to their biological counterpart: liposomes. As a result, their unique architecture makes polymer vesicles very promising candidates for nanosensors.

### Polymersomes sensing biological or chemical signals

#### pH sensing polymersomes

pH-responsive nanosensors are one of the most designed stimuli-sensitive vesicles since a lot of natural processes are highly pH-dependent, such as degradation of biomacromolecules. Moreover, vesicles sensing the decrease of the physiological pH value are very useful in cancer diagnosis, since tumor cells produce more H^+^ according to increased glucose metabolism [7].

For developing such biosensors, Quan et al. [8] encapsulated the hydrophobic fluorescent dye BODIPY into the membrane of the vesicles. The acid-sensitive dye emitted fluorescence at higher concentrations of H^+^ which allows quantitative information about the pH area. Another strategy consists in the encapsulation of fluorescent benzoxazole (BZ) molecules in the cavity of polymersomes made of self-assembled mixed polydiacetylenes (PDAs) and phospholipids [9]. In such system, FRET (Fluorescence Resonance Energy Transfer) occurred with PDA as the acceptor and the entrapped molecule as the donor. The phospholipids disrupted the vesicle structure and altered the leakage of entrapped BZ molecules due to pH changes. Consequently, the structural change of this system affected the FRET activity and impacted the overall fluorescence due to the greater distance between the donor and acceptor molecules.

**Fig. 1:**
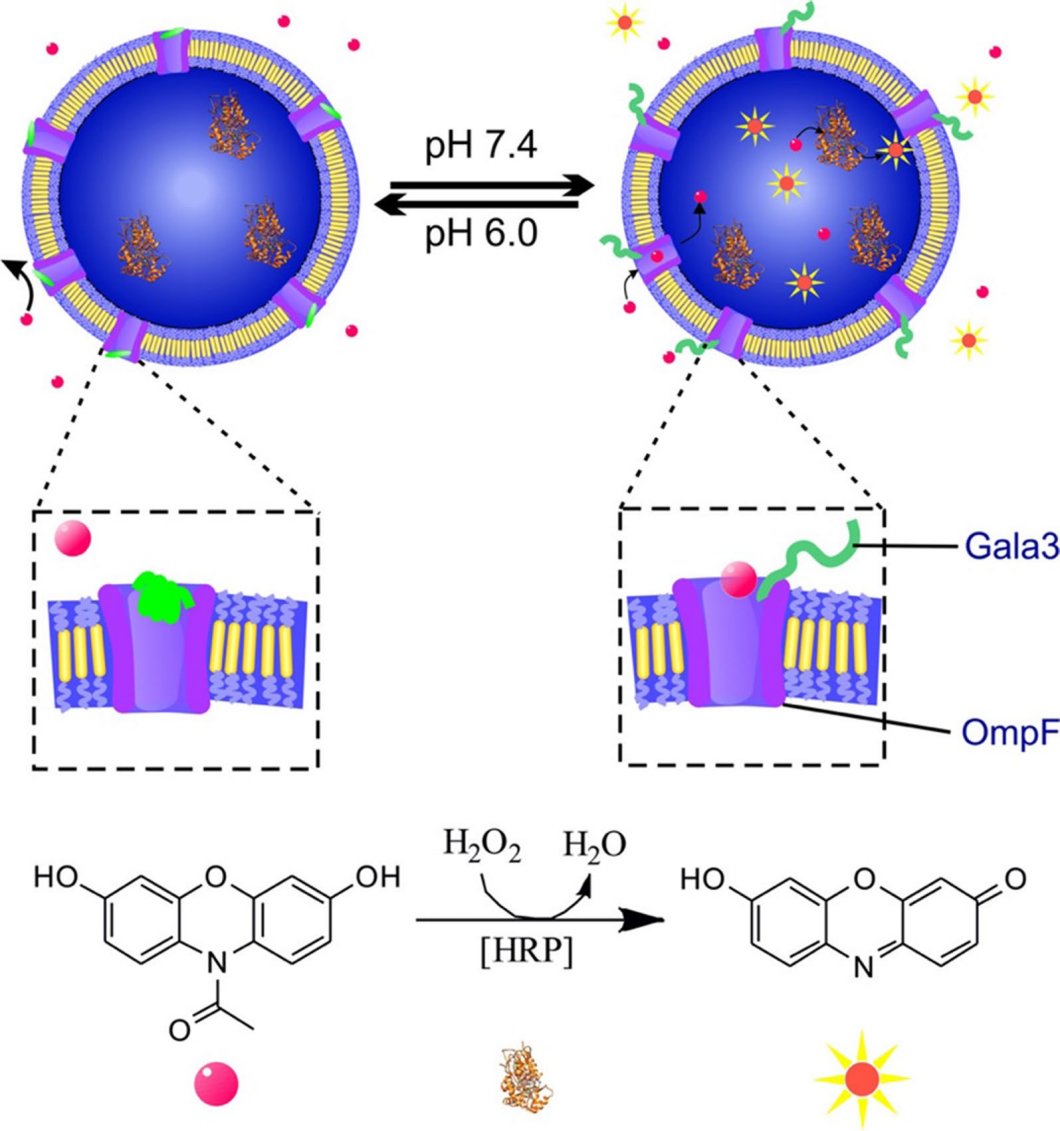
Schematic representation of a polymersome functioning by reversible pore opening and closing (left: closed state; right open state). The modified OmpF (purple; stimuli responsive group green) is inserted in the polymersome membrane. In open state Amplex Ultra Red (magenta spheres) diffused through the pore and encapsulated HRP catalyzed reaction to fluorescent product (yellow stars) which was subsequently released [10]

Also, Palivan’s group prepared Horseradish Peroxidase (HRP) loaded polymersomes with an integrated pH responsive pore [10]. The outer membrane protein F (OmpF) was modified with a 20–50 Å long peptide as molecular cap. When pH changed from 6 to 7.4, peptide charge and conformation changed significantly to allow the reversible opening and closing of the pore (Fig. 1). In its open state at pH 7.4, Amplex UltraRed passed through the pore and got oxidized by the encapsulated HRP to yield a fluorescent product.

Armes and coworkers presented a new type of polymersomes tagged with a pH-sensitive dye: a Nile Blue-based label [11]. At low pH, the protonated dye and copolymer chains remained in solution. At a pH above 5–6 the Nile-blue label became unprotonated and at a pH of 6.5, vesicles were formed because of the deprotonation and hydrophobicity of the polymer. The pH responsiveness and colorimetric shifts in the visible absorption spectrum allowed to produce a fluorescent nanosensor. Importantly, these polymersomes can be utilized for imaging pH gradients within live tumor models and intracellular microenvironments.

Recently, Craciun et al. [12] created a novel active surface that demonstrates pH responsiveness. To generate the active surface, pyranine as pH-sensitive dye was encapsulated in the polymer nano-compartments and the vesicles were attached onto glass surface. The glass surfaces were developed as pH switches able to detect both an increase and decrease in pH in the range which is relevant for evaluation of food quality.

#### Polymersomes sensing redox potential

Most reduction-sensitive vesicles contain disulfide bonds in the polymer membrane to achieve structure loss in the presence of reducing agents, like glutathione [13, 14]. Those vesicles can be applied for drug delivery since redox potentials vary highly between tumor and normal tissue. In addition to that, detection studies of the described polymersomes have already been performed successfully in cancer cells.

**Fig. 2:**
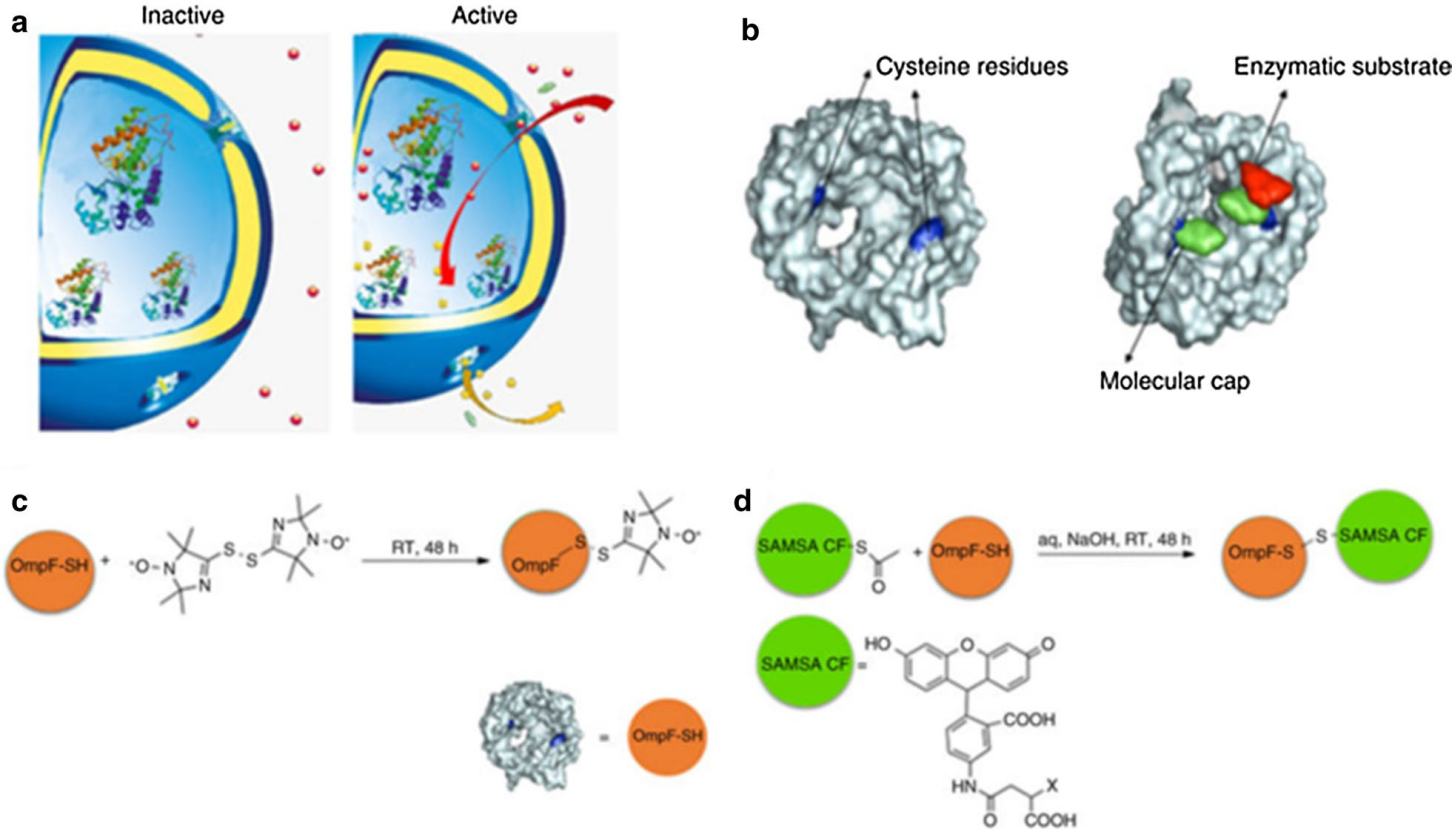
**a** Schematic representation of modified OmpF acting as a reduction-sensitive gate in catalytic nanocompartments. **b** Molecular representation of the OmpF-M cysteine mutant with and without molecular cap. Chemical modification of OmpF-M cysteine mutant with bis(2,2,5,5-tetramethyl-3-imidazoline-1-oxyl-4-yl) disulphide (**c**) with the fluorophore SAMSA-CF (**d**) [15]

Recently, Palivan’s group developed artificial organelles which enabled the detection of changes in glutathione concentrations [15]. Protein gates were inserted in the membrane of reduction-sensitive polymersomes containing HRP (Fig. 2). The inserted protein gates were engineered by attaching molecular caps to genetically modified channel porins in order to induce redox-responsive control of the molecular flow through the membrane. In its open state where the reduction-sensitive molecular cap was cleaved from the pore, Amplex UltraRed could pass through the pore and HRP catalyzed the reaction to give the fluorescent product resorufin. Additionally, those nanosensors are functional in a vertebrate ZFE (zebrafish embryo) model, which proves that the concept of artificial organelles as cellular implants is feasible in vivo.

#### Polymersomes detecting SO_2_ and biomolecules

Many different compounds and molecules can act as bioactive signals as hydrogen peroxide (H_2_O_2_), dioxygen and adenosine triphosphate (ATP) to name but a few. Several strategies using sensitive polymersomes were developed for their detection. Vesicles produced through the assembly of conjugated polydiacetylene is one of them. Polydiacetylenes have been well studied for their chromatic-transition properties and have been successfully employed for the development of colorimetric biosensors since the polydiacetylenic structures can change color with environmental perturbations [16–19].

Recently, polymersomes made of PDA were reported for the detection of α-cyclodextrin, which is produced during the enzymatic degradation of starch [16]. In this study, azobenzene-containing PDA vesicles have been used for photo-controlled inclusion and exclusion of α-cyclodextrin. The resulting perturbation of the artificial vesicle membrane could be visualized thanks to a blue to red color change. In addition to that, Ma et al. created PDA vesicles which used the energy transfer between the encapsulated fluorescent dye BODIPY and the PDA backbone to detect an organic amine, triethylamine, which caused structural changes of the vesicles and thus, a color transition from blue to red could be observed [17]. Wang et al. [18] developed a new colorimetric method for detecting oligonucleotides. Crosslinked PDA vesicles were functionalized with probe DNA. This sensing method was based on interaction between the probe DNA and the targeted DNA. The amplification tag recognized linear oligonucleotides and the structural change of PDA due to the oligonucleotide could be detected by color transition from blue to red.

PDA-based vesicles were also used to develop H_2_O_2_ sensors. This is particularly interesting considering the involvement of this molecule in many naturally occurring key processes. For instance, H_2_O_2_ sensors could be further exploited for food or environment monitoring. Polydiacetylene vesicles functionalized with phenylboronic acid were developed as an optical sensing method [19]. A color change is observed when the polymerization of PDA is initiated by radicals generated from the catabolization of H_2_O_2_ by the enzyme. Even if this system is not extremely sensitive, the color change was enhanced by the presence of phenylboronic acid.

**Fig. 3:**
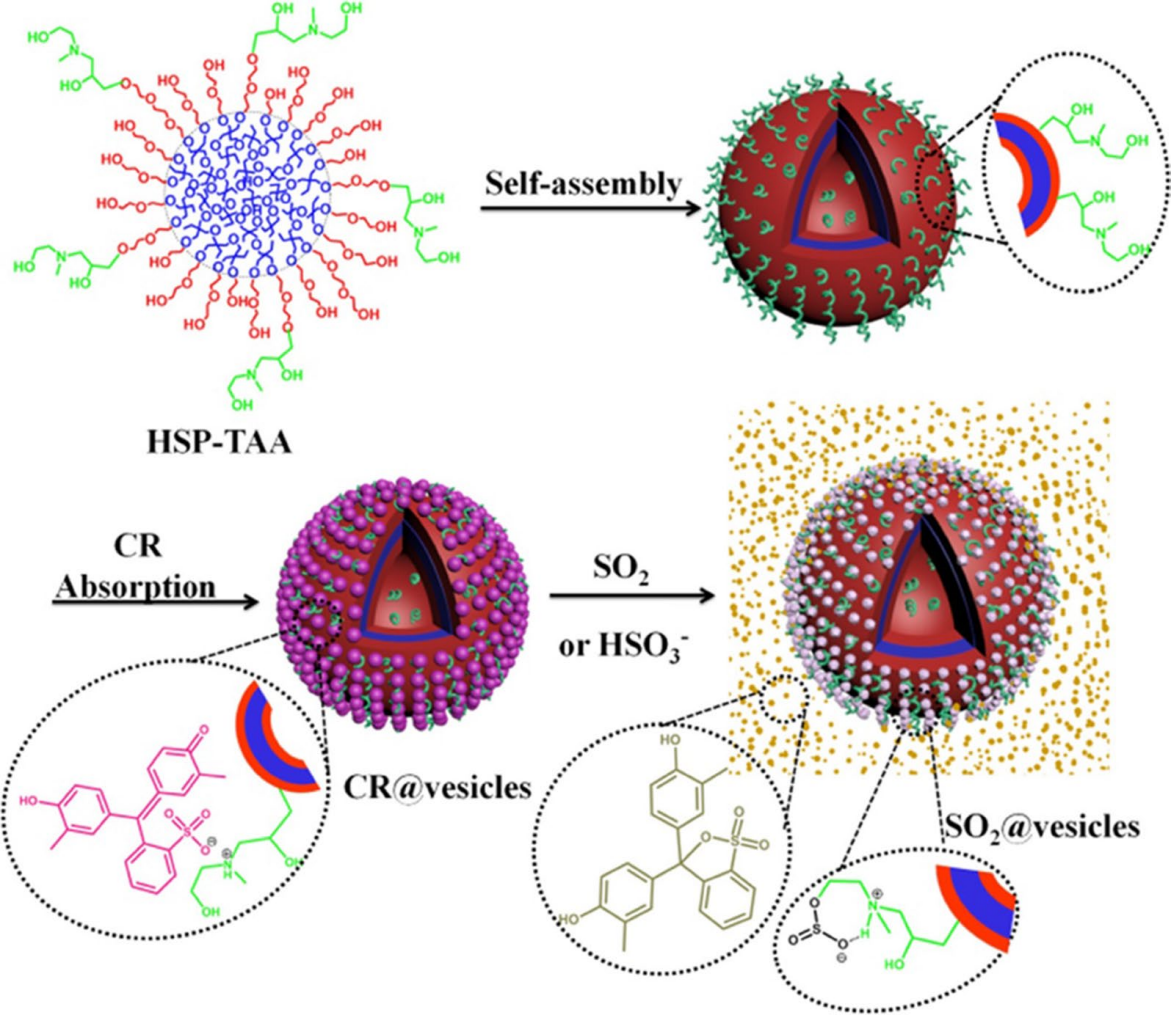
Illustration shows the main concept of SO_2_-sensing and self-assembly of the vesicles with functionalized surface. The hydrophobic HBPO core of the sensor is blue, the hydrophilic PEO arms are red. The TAA groups (green) undergo proton exchange with cresol red and cresol red-immobilized vesicles (purple) are formed. In the presence of SO_2_, the protonated yellow cresol red is released [20]

A different class of colorimetric sensors for small molecules was developed by Huang et al. They described the first polymer vesicle sensor for the visual detection of sulfur dioxide (SO_2_) and its derivatives among ions in water [20]. For their study, a strong binding ability between tertiary alkanolamines (TAA) and SO_2_ has been used as the driving force for detection (Fig. 3). Vesicles were formed using amphiphilic hyperbranched copolymer composed of hydrophobic poly(3-ethyl-3-oxetanemethanol) (HBPO) core and linear poly(ethylene oxide) (PEO) arms terminated with TAA groups. By addition of cresol red, TAA groups located at the surface of the vesicles underwent proton exchanges with these dyes, leading to purple vesicles. Then, upon the presence of SO_2_ and its derivatives, TAA reacted with them and colorless vesicles were formed, followed by the release of protonated cresol which is yellow.

In addition, a polymersome-based sensor for adenosine triphosphate (ATP) has been developed by Liedberg and coworkers [21]. They encapsulated an enzyme (alkaline phosphatase) and a fluorescent reporter polymer (poly-1-(3-((4-methylthiophen‐3‐yl)oxy) propyl)quinuclidin‐1‐ium) into vesicles formed with amphiphilic di-block copolymer (polystyrene‐*b*‐polyisocyanoalanine(2‐thiophene‐3‐yl‐ethyl)amide). Exogenous ATP could passively diffuse through the membrane and quenched the reporter polymer. Then, alkaline phosphatase hydrolyzed the reporter bound-ATP leading to partial recovery of its emission. Such system could be considered as a model for processes involving accumulation/consumption of ATP inside discrete vesicular compartments.

Moreover, several ions such as K^+^, Na^+^ or H^+^ can be detected by polymersomes formed from a library of different poly(2-methyloxazoline)-*block*-poly(dimethylsiloxane)-*block*-poly(2-methyloxazoline) (PMOXA_x_-PDMS_y_-PMOXA_x_) triblock copolymers, thanks to a particular ion selective permeability. The insertion of gramicidin (gA) peptides in their membrane formed biopores that permit different ions to enter inside the vesicles cavities. This exchange across the membrane can be visualized through the encapsulation of dyes that are specific to these compounds: changes in fluorescence intensity are observed upon the entrance of these small molecules inside polymersomes [22].

Also, Zhang et al. [23] developed an “active surface” serving for efficient detection of sugar alcohols based on immobilized protein-polymersome nanoreactors. These sensors showed high sensitivity due to the rapid change in the fluorescence intensity of the surface in the presence of sugar alcohols. The detection method based on polymersomes enabled a selective passage of sugar alcohols through the synthetic membrane. Encapsulated enzymes (ribitol dehydrogenase) in the polymersomes were used as biosensing entity.

Hammer’s group focused on the development of a nano-biosensing platform made of patterned microfluidic synthesized cell-sized polymersomes that are immobilized on a surface [24]. These microarrays are developed using giant polymersomes that are functionalized with biotin and organized using micropatterned islands of NeutrAvidin. These polymersomes can detect the presence of a range of soluble molecules of interest added to the array by capturing these compounds on their membrane, leading to a change of fluorescence. This system provided numerous advantages as polymersomes are patterned and functionalized at the single vesicle level. This led to a better uniformity of diameter, higher loading efficiencies and a relatively simple but controllable organization of the vesicles on the surface.

#### Enzyme-sensing polymersomes

**Fig. 4:**
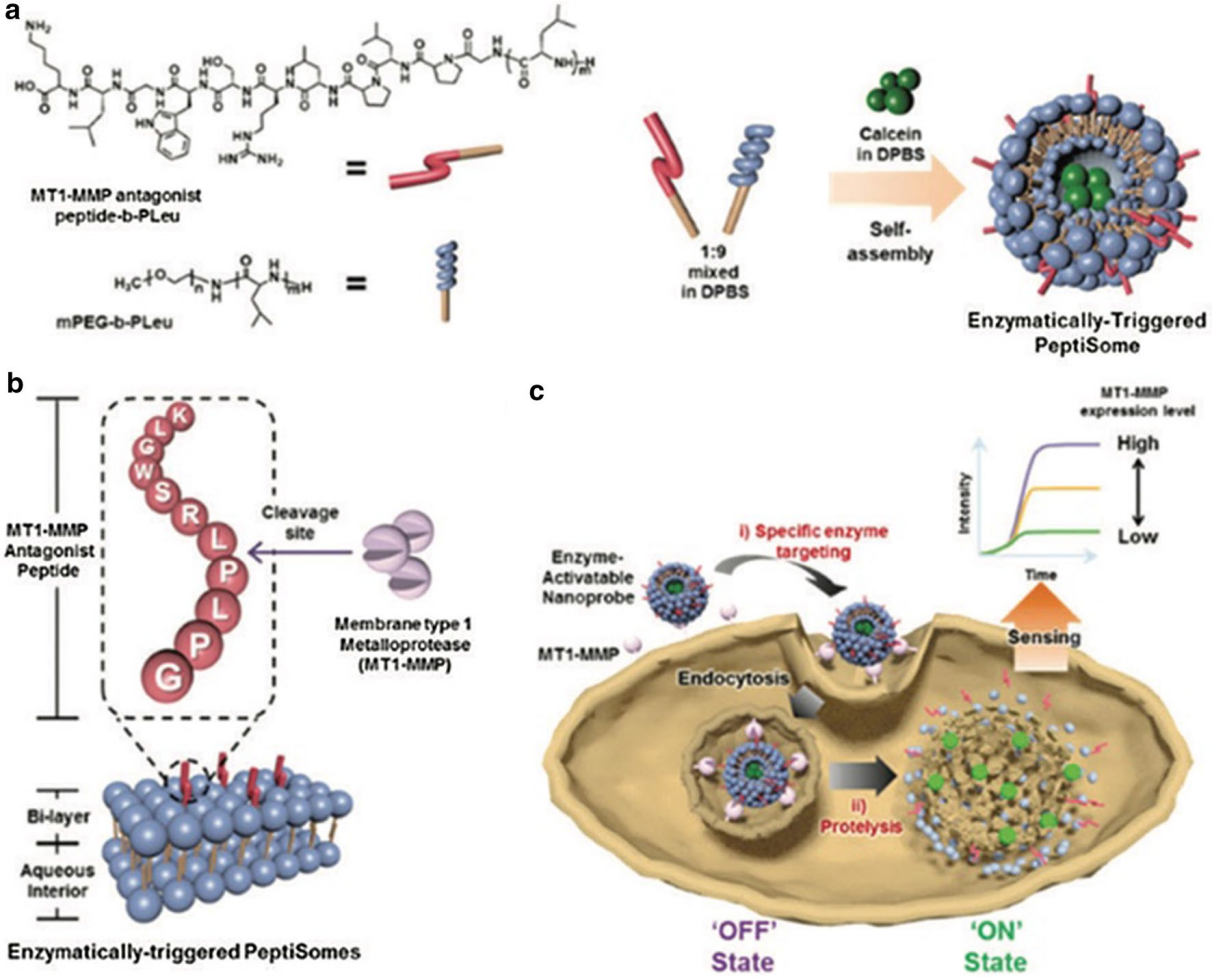
**a** Preparation of the calcein-loaded PeptiSome with methoxy-poly(ethylene glycol)-block- poly(rac-leucine) (mPEG-b-pLeu) mixed with MT1-MMP antagonist peptide-b-pLeu, then with calcein. **b** Chemical structure of the peptide sequence in the PeptiSome bi-layer membranes. The cleavage site is located between Phe and Leu. **c** Schematic representation of calcein-loaded PeptiSome and enzyme-activatable nanoprobe. When the calcein is loaded into PeptiSomes, fluorescence is self-quenched. After PeptiSomes enter cancer cells by endocytosis, enzymatic cleavage of the peptide by MT1-MMP releases calcein, which is no longer quenched [25]

Enzymatic sensors cover a wide range of applications and can be used to detect special types of cells (for example, tumor cells), pathogenic micro-organisms or even genetically modified organisms. Kim et al. developed biohybrid polymersomes that respond to matrix metalloproteinase type 1 (MT1-MMP1) which plays an important role in metastasis associated-cancer cell trafficking [25]. The sensitive polymersomes were prepared with membranes based on methoxy-poly(ethylene glycol)-block-poly(rac-leucine) (mPEG-b-pLeu) and MT1-MMP1 antagonist peptide (activatable binding moiety)-b-pLeu (PeptiSome) (Fig. 4). Moreover, their calcein-loaded PeptiSome-based approach exploited dye release from a capsule whose wall is selectively cleaved by MT1-MMP and enables detection of tumor cells.

Other enzyme-responding polymersomes are promising candidates for targeting bacterium. Haas et al. created a new hyaluronidase-sensing system based on hyaluronic acid and polycaprolactone that can be assembled into polymersomes by inversed solvent shift method [26]. The triggered release of encapsulated dye enables an autonomous detection of hyaluronidase which is produced by *Staphylococcus aureus* bacteria. Another group also focused on the detection of *Staphylococcus*, using PDA vesicles coupled to specific antimicrobial enzyme lysostaphin [27]. A color transition and an enhancement of the fluorescence is observed when the enzymes interact with bacteria. The efficiency of this system is also enhanced as the vesicles are immobilized on a channel mimicking a fish-gill structure. This set up increase the surface-to-volume ratio, which maximize the interactions of the enzymes with bacteria located in the fluid running through the channel. Plus, this system also allows antimicrobial effect as the enzyme used for the detection of these bacteria is also antimicrobial. Jung et al. [28] developed a colorimetric biosensor based on polydiacetylene vesicles (PDA) that detect phosphinothricin acetyltransferase (PAT) which is an important marker enzyme of genetically modified crops. Immuno-hydrogel beads are formed by encapsulating anti-PAT conjugated PDA vesicles in poly(ethylene glycol) diacrylate hydrogel matrix, in order to increase PDA vesicles sensitivity and robustness. Following the immunoreaction, a clear color change was observed.

### Polymersomes based sensors for physical changes

Physical phenomena provide extremely convenient usage in vivo as they are deeply penetrating the tissue, relatively safe and easy to use. In that regard, a lot of studies have been focused on the development of responsive polymersomes to physical changes (temperature, light, ultrasound, magnetic field…) [29–32]. In fact, such system can provide interesting options for drug delivery, imaging or therapy as the release of the encapsulated compound could be generated by a physical trigger. Nonetheless, far less polymersomes sensing physical changes have been developed compare to their equivalent polymersomes sensing biochemical signals.

**Fig. 5:**
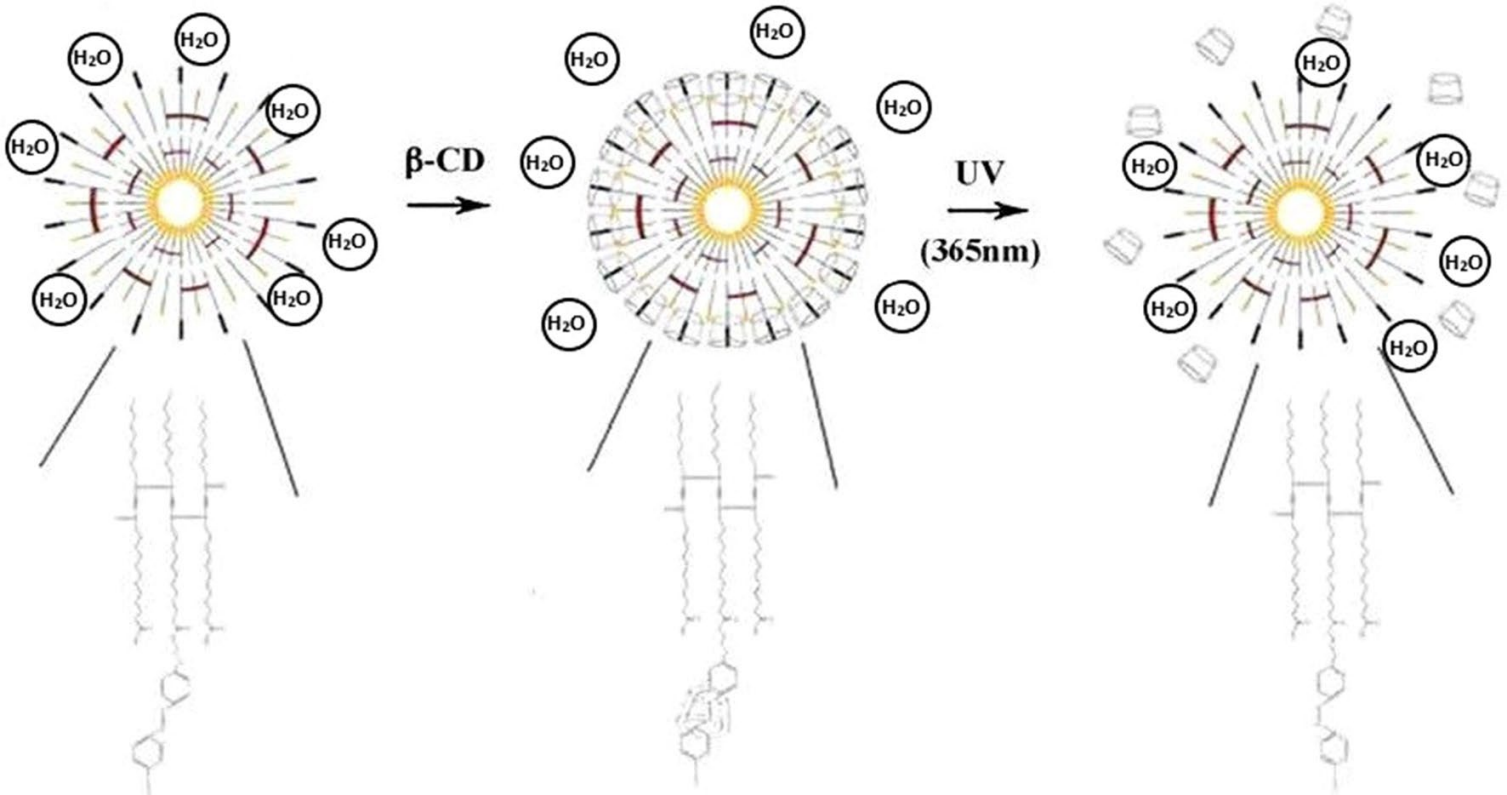
Illustration of the photo-controlled inclusion or exclusion reactions of β-cyclodextrin with the azobenzene-containing polydiacetylene vesicles [33]

Chen et al. [33] reported a temperature sensor based on vesicles made of azobenzene-containing polydiacetylene (PDA). These polymersomes showed linear variations of their fluorescence intensity depending on temperature. The resulting fluorescence signal was significantly enhanced by the addition of β-cyclodextrin (β-CD). In fact, an external photo-stimulus triggered the inclusion or exclusion of β-CD within the PDA assemblies, leading to disruption of the ordered structures of PDA (Fig. 5). The resulting inclusion complexes forced the colour transition in a selective and predictable way. Then, this system allows the detection of temperature variations ranging from 25 to 80 °C and also showed good biocompatibility. Therefore, such polymersomes could be potentially used as temperature sensors in chemical or bio-environment.

## Planar polymer membranes based nanosensors

### Introduction

Planar polymer membranes nanosensors are seeing a constant increasing interest and offer undoubted advantages over conventional analytical methods providing quicker and essential information. Different methods to develop distinctive planar polymer membrane based nanosensors have been established, but still effort has to be made in order to improve their performance. Polymer based nanosensors allow us now to probe numerous chemical or biological analytes, from gas, ions to more complex structure like protein or oligonucleotides. In that regard, the polymer membrane will play a major role in the recognition efficiency of the sensing device. Planar polymer membranes can be used to immobilize active species or improve the sensitivity through an improved signal transduction. In all cases the polymer membrane is either designed or chosen to provide an appropriate signal or improve the sensing device efficiency. This part of the review will focus mainly on the planar polymer membranes of the nanosensor and their characteristic features as material of sensors, whether they serve as recognition material, used as part of the transduction mechanism or operate as intermediate mechanical support or pattern. We cover the topic under four main sections, multiple components polymer membranes, molecularly imprinted polymer membranes, conducting polymer membranes and nanoporous polymer membranes. Certain polymer membranes can belong to several class of polymer, for instance, a conducting multiple components polymer membrane.

### Multiple components polymer membranes

**Fig. 6:**
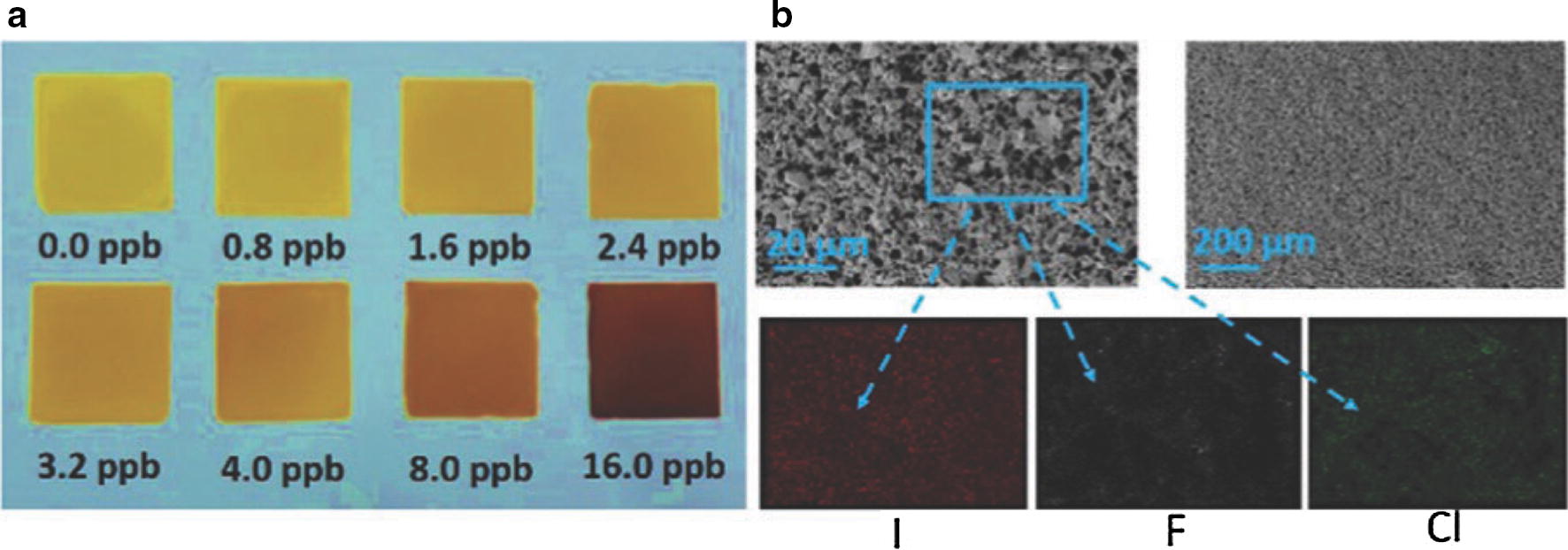
**a** Colour change of the Cu_4_I_4_–MOF-based MMM exposed to gaseous HCl with different concentrations (< 1 min). **b** SEM image of the surface of MMM at different magnitudes after exposure to gaseous HCl and the EDS mapping spectra of the area as labelled [35]

Mixed matrix polymer membranes (MMMs), composite membranes or hybrid organic–inorganic membranes, all refer to the same type of polymer membranes, they are all multiple components polymer membranes. Such membranes have been developed to overcome limitations encounter with conventional organic or inorganic materials. In those types of membranes, the polymer is rarely the sensing material but rather the material in which the organic or inorganic element is embedded. In MMM based sensors, polymer membranes can be designed to support different types of recognition elements, from the simple chemical probe as inorganic particles to the more complex ones as nucleic acid, enzyme, antibodies or cells [34]. For instance, Zhao et al. [35] reported a Cu_4_I_4_-metal organic framework (MOF) based mixed matrix membrane for gaseous HCl sensing. The polymer membrane was prepared by one-step in situ self-assembly of a tri-armed oxadiazole-bridged ligand and CuI in a polyvinylidene fluoride polymer binder solution. A clear colour change was observed from light yellow to dark red when the membrane was exposed to different concentrations of gaseous HCl, (Fig. 6). The change was due to the replacement of iodine by chloride into the framework. This MOF-MMM also exhibited faster gaseous HCl sensing than free micro-sized Cu_4_I_4_-MOF, demonstrating the beneficial gas permeability of the MMM.

Another example of polymer-MOF composite membrane was developed by Sachdeva et al. [36] in order to be used as nanosensor for the detection of alcohols but more broadly for a range of gaseous analytes. Commercial Matrimid polymer was chosen as the polymer matrix and nanoporous aluminium nanoparticles were imbedded to enhanced the intrinsic affinity of analytes for the polymer membrane. They observed an increase by a factor of two of the capacitive response when the nanosensor was exposed to methanol compared to a bare Matrimid film. Such material is easily integrated and compatible with existing fabrication techniques in the field of microelectronic [3].

**Fig. 7:**
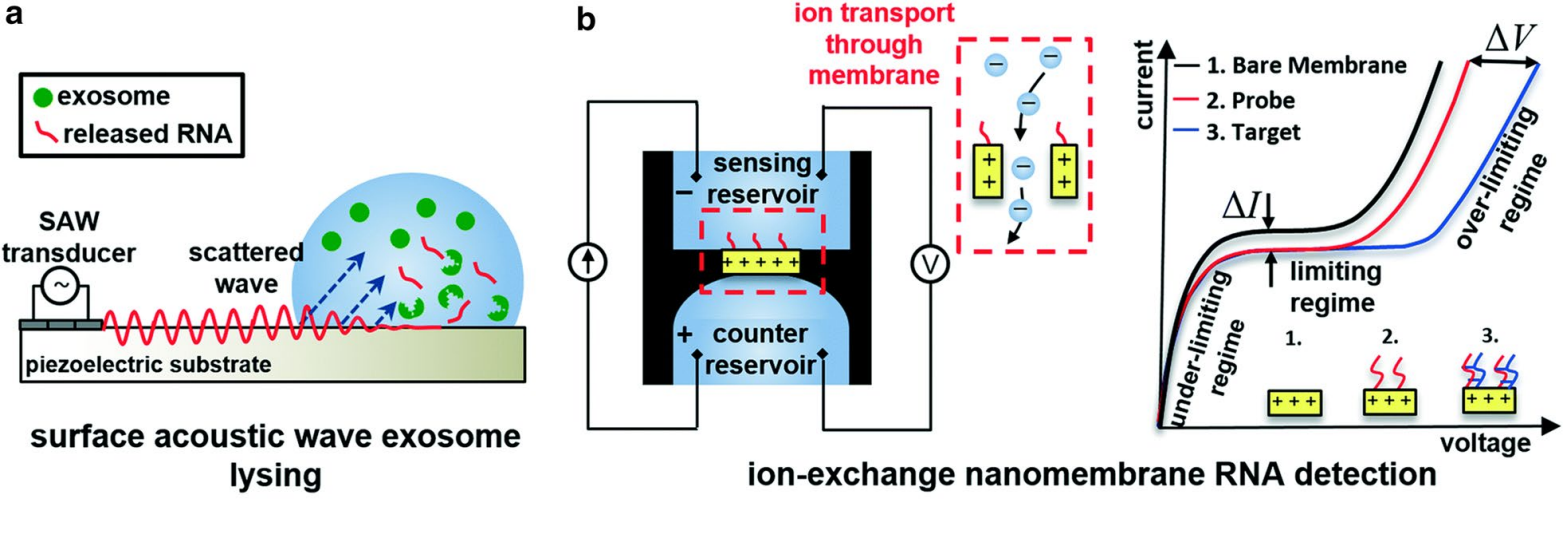
**a** Schematic of SAW device (side view) and SAW-induced lysing of exosomes to release RNA for detection. SAWs generated at the transducer refract into the liquid bulk, inducing fluid motion, and electromechanical coupling also generates a complimentary electric wave at the surface of the substrate. **b** Schematic of ion-exchange nanomembrane sensor consisting of two reservoirs separated by the membrane. RNA in the sensing reservoir hybridize to complimentary oligos immobilized on the surface of the membrane. The inset shows the ion transport through the device to generate current and the right image is a characteristic current–voltage curve illustrating the under-limiting, limiting, and over-limiting regimes [38]

Numerous multicomponent membranes are used for biosensors [34]. For instance, to develop a sensor for the detection of nucleic acid, Senapati et al. used an ion-exchange nanomembrane [37]. The nanomembrane was made of divinylbenzene/polystyrene particles embedded into a polyethylene-polyamide/polyester matrix. The polyethylene acts as a binder and the polyester/polyamide fibers provided the mechanical stability for the membrane. Specific oligoprobes were then attached covalently on the surface. The sensor works on the following principle: the hybridization of the molecular oligoprobes with the targeted nucleic acid molecules alter the ion conductance across the membrane solution that results in a significant shift in the recorded current voltage characteristic (CVC). The same group developed a sensor for exosomal ribonucleic acid (RNA) for pancreatic cancer study and diagnosis [38]. They used the same type of ion exchange nanomembrane and the same kind of detection method than previously cited where RNA gets hybridized to complementary oligonucleotides probes immobilized on the surface of the membrane. CVC are then recorded and linked to the detection of targeted RNA, (Fig. 7). In that case, a surface acoustic wave (SAW) device was used to generate RNA release from exosomes.

### Molecularly imprinted polymer membranes

Among the several types of sensing materials using polymer membranes, the molecularly imprinted polymer (MIP) membrane is one of the most specific materials, as it requires the imprint of the analyte itself to develop the sensing material. Other methods with specific receptors as enzymes, microbes or antibodies show high selectivity towards their analytes but are often more expensive with a poor chemical or temperature stability [39, 40]. MIPMs offer several advantages as high surface area, a wide panel of analytes, large number of recognition sites and a good thermal and chemical stability. Zhang et al. [41] developed an alternative method for the fabrication of potentiometric sensors based on MIP membranes. They produced at high temperature a soluble MIP, used as receptor, that they incorporated into a plasticized polymer membrane. They showed an increased sensitivity for bisphenol AF compared to classical MIP based sensors. The membrane also exhibited a lower detection limit of 60 nM. The advantage of such method lies in the flexibility towards the choice of the MIP membrane. Such imprinted material has been also used for toxic compound detection. Zhang et al. [42] developed a nanoscaled MIP membrane that was constructed for the selective detection of herbicide simazine (SMZ). The electrochemical sensor was built through the self-assembly of *o*-aminothiophenol (ATP) and the electropolymerization of *o*-aminothiophenol functionalized gold nanoparticles (ATP@AuNPs). The incorporation of gold nanoparticles has been proved to increase the sensitivity towards simazine compound by 23 times, compared to a bare gold electrode. The rigid structure formed from ATP@AuNPs electropolymerization also improved the selectivity of the SMZ imprinted sensor.

The same method was also applied for the detection of endocrine disruptors in different media [43]. For instance, Yuan et al. [44] selected 17β-estradiol (E2) as target analyte because of the intense disequilibrium changes in immune, cardiovascular and nerve system that it can cause. An electrochemical nanosensor based on a MIP membrane to detect E2 was constructed through the self-assembly of 6-mercaptonicotinic acid (MNA) and E2 on a glassy electrode. The latter was first modified with platinum nanoparticles. The subsequent electropolymerization led to the construction of MIP membranes with high selectivity and sensitivity towards E2, even in water samples where the sensor demonstrated high efficiency among several interferences.

### Conducting polymer membranes

The class of “conducting polymer” includes several types of material with electronic or ionic conductivity as well semi-conducting polymers, doped conjugated polymers, redox polymers, polymer composites and polymer electrolytes. The great design flexibility of conducting polymers make them a polymer of choice for different type of sensors and are broadly used in chemical or biochemical sensors [45–47].

**Fig. 8:**
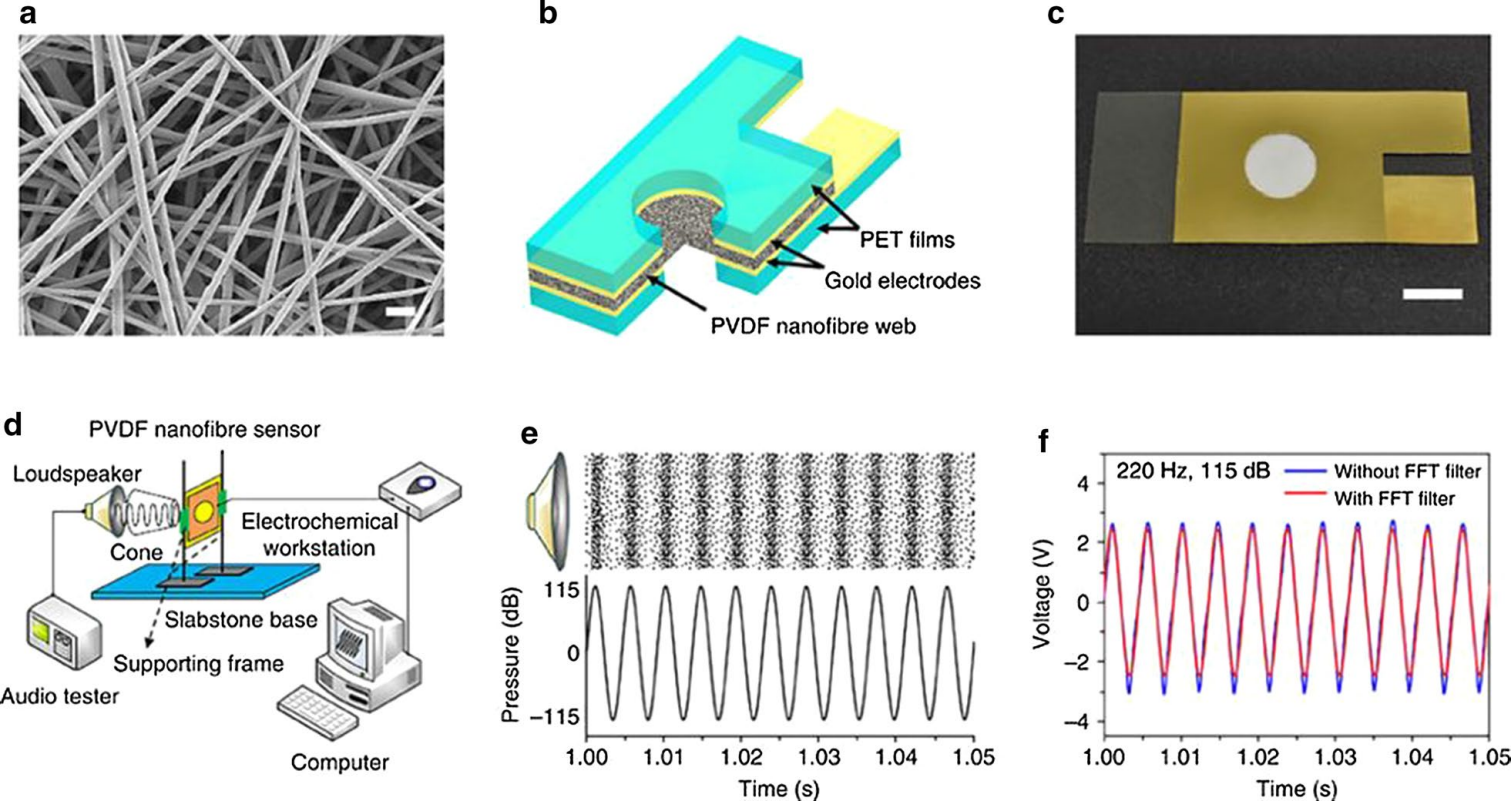
**a** SEM image of the PVDF nanofibres (scale bar, 1 μm), **b** schematic illustration of sensor structure, **c** digital photo of the device (scale bar, 1 cm), **d** schematic illustration of the setup for testing the sensor device, **e** illustration of sound wave (the black dots illustrate the motion of air molecules associated with sound), **f** voltage outputs of the device under sound with and without FFT treatment (hole diameter, 12.8 mm; web thickness, 40 μm; web area, 12 cm^2^) [48]

The structural characteristics of the polymer membrane is sometimes as important as the polymer itself. For example, Lang et al. [48] showed that assembly of nanofibers into a polymer film was much more effective than a regular commercial dense film. They produced a sound sensor using a piezoelectric poly(vinylidene fluoride) (PVDF) film made of nanofibers. The acoustic sensor was made by placing two transparent terephthalate films that were gold-coated, on each side of a PVDF film containing the nanofibers. The gold coated part was contacted with the PVDF film and they functioned as electrodes to collect electrical signals. They showed that the piezoelectric nanofibers contained in the polymer film prepared from electrospinning exhibited great acoustic-to-electric conversion capability and were able to detect low frequency sound with a sensitivity of 266 mV Pa^−1^ (Fig. 8).

**Fig. 9:**
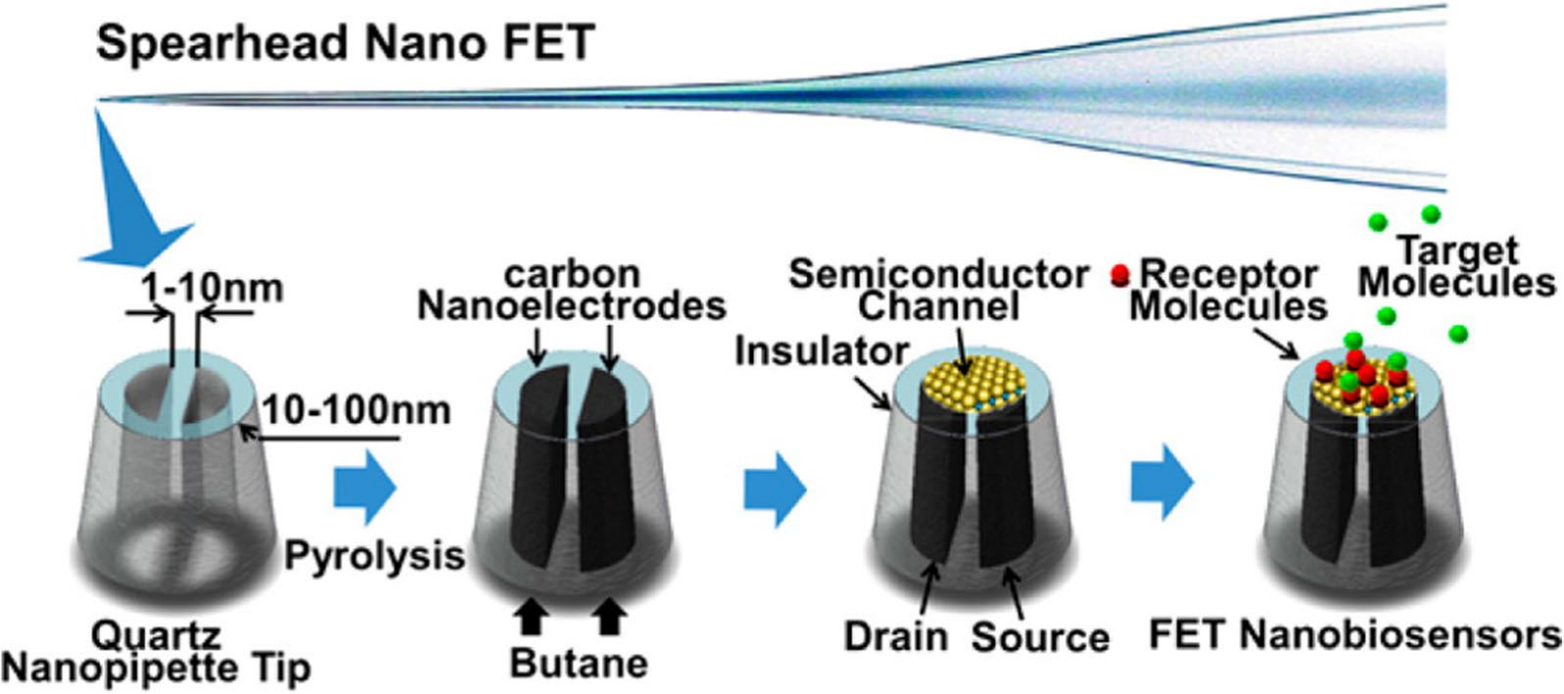
A nanometer-scale field-effect-transistor is created by depositing a thin layer of semiconductor material on the tip of spear-shaped dual carbon nanoelectrodes fabricated by pyrolytic decomposition of butane inside nanopipettes. The two individually addressable electrodes serve as drain and source. Immobilising suitable recognition biomolecules on the semiconductor transistor channel yields selective FET biosensors [49]

Zhang et al. [49] built a nanobiosensor that could be used for single cell analysis. They created a nanometer scale field effect transistor (FET) by depositing a thin film of a semiconducting polymer, polypyrrole (PPy), on the tip of a spear-shaped dual carbon nanoelectrodes. Then, they used hexokinase, an enzyme that catalyse the addition of phosphate from ATP in the glycolysis reaction, to be immobilized on the semi conducting polymer and yield a selective FET nanobiosensor (Fig. 9). This device could be used to detect other analytes than protons if the chemical conversion of the analyte can be translated into a pH change.

In the same way than multiple component polymer membranes, conducting polymer membranes can be tuned to mesh with an alternative material and to improve sensitivity and selectivity of the conductive material. The counter part of the organic polymer can be a metal oxide, a metal, an organic material or carbon nanotubes, to name but a few [50]. Mahato et al. [51] developed a sensor material with high sensitivity towards aliphatic alcohols. A poly(*N*-[4*H*-1,2,4-triazol-4-yl]acrylamide) (PNTA) polymer was synthesized and blended with a poly(vinyl chloride) (PVC) to form a membrane. The polymer membrane was then incorporated to an electrode and its stability and response to aliphatic alcohols were recorded. Measurements showed stability up to 1000 s for six different aliphatic alcohols, as well as a good discrimination ability towards these short chain aliphatic alcohols.

**Fig. 10:**
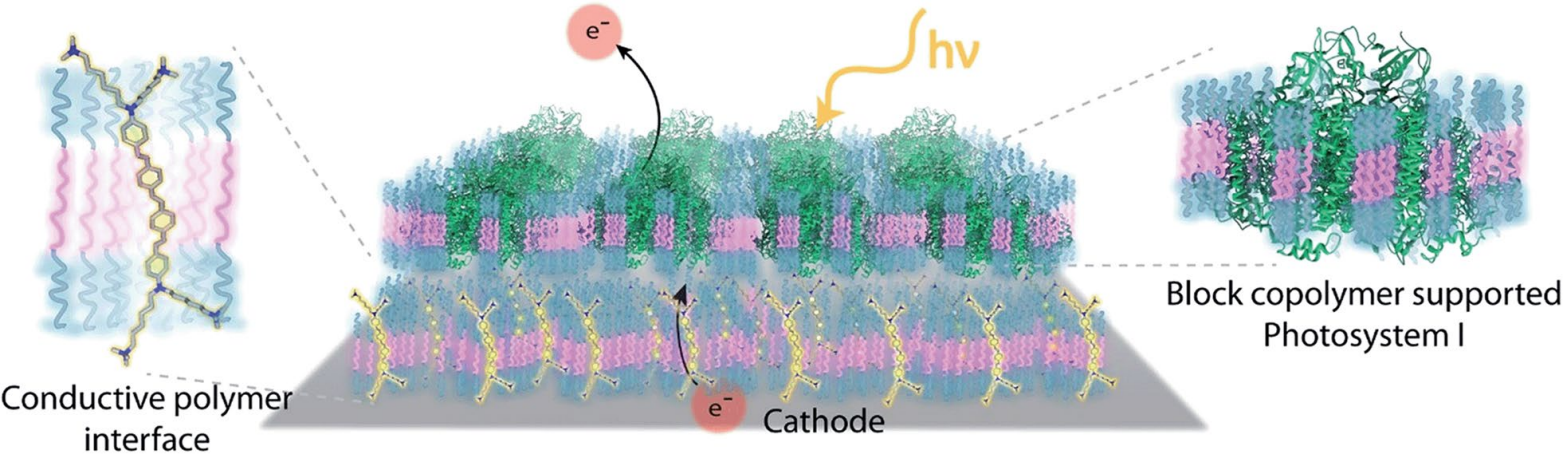
Design of Photosystem I (PSI) block copolymer integrated membrane. A block copolymer (BCP) bilayer membrane based interface with conjugated electrolytes (COEs) provides efficient electron transfer to Photosystem I (PSI) proteins incorporated at high density in another block copolymer membrane. COEs and the photosynthetic membrane protein, PSI, were stabilized in poly(butadiene)_12_-poly(ethylene oxide)_8_ (PB_12_-PEO_8_) BCP membranes. Short-chain amphiphilic BCP membranes can have similar thickness as lipid bilayers (~ 4 nm). Hydrophobic regions (pink) represent the PB block of the BCP and stabilize the hydrophobic region of COEs and the protein surface. COEs intercalated into a BCP bilayer form two-dimensional membranes on electrodes and enable efficient electron transfer to the proteins, which allows large photocurrent generation. This hydrated film provides a biocompatible environment to the protein components protruding from the BCP membrane, in the absence of which could lead to protein denaturation on un-functionalized metal electrodes. The hydrophilic blocks (blue regions) of the BCP are PEO blocks and are hydrated with water, making them compatible with amino acids located near the membrane interface. The hydrophobic interactions between membrane proteins, such as PSI, and BCPs lead to large, self-assembled planar membrane structures in water. The photosynthetic protein functionalized membranes can then be integrated into a bioelectronic device using electrostatic interactions to immobilize PSI membranes on the COE intercalated BCP bilayer. Light energy collected by the PSI protein pumps electrons from the electrode to the solution phase [52]

In biosensors where electron transfer machinery is exploited at electrode interfaces, development of polymer membranes that insure proper integration of protein to electrodes to maximise electron transfer, is of great importance. In that regard, Saboe et al. [52] developed a system where a conducting bilayer block-copolymer membrane was used in combination with a photosystem I protein and described the first application of membrane proteins stabilized in block-copolymer support for an electrochemical device. The first block-copolymer, poly(butadiene)_12_-poly(ethylene oxide)_8_, with an integrated conjugated oligoelectrolyte acted as conductive interface that provided efficient electron transfer to the photosystem I and the second block-copolymer was used to stabilize the protein. After functionalization of gold electrodes with the conductive bilayer membranes, they reported a photocurrent approaching 35.0 μA cm^−2^, which was among the highest observed so far for such system on a per protein basis (Fig. 10).

### Nanoporous or single channel polymer membranes

Nanoporous polymer membranes have been used for several sensing purposes. For example, they can be used to sense humidity. This ability is of great interest for a wide range of applications such as electronics processing, air conditioning or meteorological systems. For instance, low-cost humidity sensors were developed by Yang et al. [53] using polycarbonate, cellulose acetate or nylon. In this paper, the authors proposed two different methods to fabricate the nanopore-based polymer humidity nanosensors. The first method implies the use of an adhesive paper and the second the use of a silicon mask. Humidity level was determined through the resistance or capacitance recorded between electrodes deposited on the nanoporous polymer membranes. Different designs were tested for the nanosensors and the lowest sensitivity value recorded among the different designs was three orders of magnitude higher than the maximum sensitivity achieved with other humidity sensors such as nanoporous alumina or nanoporous silicon-based humidity sensors which prove the efficiency of such material [54, 55].

#### Block copolymer as nanopattern for nanosensors

One of the main advantages of block copolymer over homopolymer is its possibility to selectivity sacrifice (or anneal) one part of the polymer without affecting the assembly or organisation of the other block. They offer unique morphologies or ability to form nanopores [56]. BCP can be used as pattern for nanofabrication because of their specific features and good self-assembly capacity. The creation of specific patterns with block copolymers represents an affordable and straightforward method to be applied in various nanotechnology and has been the subject of interest in gas sensing applications. Guo et al. [57] reported the fabrication of 3D gyroidal networks of a block copolymer template containing gyroidal nanopores. They precisely controlled the thickness of the layer deposition varying the number of atomic layer deposition cycles and formed ZnO nanorods and nanotubes. The block copolymer template allowed the formation of nanomaterial that can act as gas sensing. They were shown efficient for ethanol and formaldehyde sensing. Bas et al. [58] produced an electrochemical sensor of hydrogen peroxide using block copolymers templated iron oxide nanopatterns. A polystyrene-block-polyvinyl polymer was used because of its electron-donating character and was spin coated on a functionalized indium tin oxide surface. The surface was then solvent annealed to led to a nanoporous structure that was then reconstructed through iron nitrate inclusion. The final step consisted in an UV/ozone treatment that removed the polymer matrix to yield the iron oxide nanodots. This enzyme-free sensor showed low detection limit and high selectivity and sensitivity towards H_2_O_2_ with a detection limit of 1.1 × 10^−3^ mM.

#### Sensing by volume exclusion effect

Polymer biochemical sensing membranes are based on the same principle than biological ion channels. There are mainly two types of detection signal that rely on the variation of ionic current in channels: sensing by volume exclusion effect and sensing by electrostatic effect. In nanopore sensing, the transmembrane ionic current is proportional to the pore size. The crossing of an analyte through the nanochannel decreases the cross-section and consequently affect the current signal measured. The measured current signal reflects then the size and shape of the molecule [59, 60]. For example, in conventional nanopore based DNA sensors, the short translocation time lead to low resolution and accuracy. Meller and coworkers produced a nanopore-nanofiber based membrane to detect double-stranded DNA capable of slowing the translocation speed by two orders of magnitude [61]. This method enabled greater temporal nanopore resolution and greater discrimination among DNA lengths. The membrane was made by electrospinning copolymer blends of poly(e-caprolactone) and poly(glycerol monostearate-co-e-caprolactone) onto the nanopore membrane. Many fields as sequencing, gene expression or genotyping may benefit from such method using polymer coating to control the biomolecule translocation [62].

#### Sensing by electrostatic effect

In the second type of polymer biochemical sensing membrane, the sensing property is mainly based on electrostatic effect. The inner wall of nanochannels is functionalized with distinct recognition molecules depending on the targeted analyte [63]. Ali et al. [63] used a similar method and reported an alternative approach to incorporate biosensing elements into polyethylene terephthalate polymer nanochannels membrane by using electrostatic self-assembly to produce streptavidin nanosensors. A bifunctional positively charged macromolecular multivalent ligands made of biotinylated poly(allylamine) was used to interact with the negatively charged carboxylic groups of the pore surface and exposed the binding side inside the nanopores without restraining their recognition properties. Then the addition of a streptavidin solution into the nanopores led to specific bindings with the biotin ligand. This approach allowed a higher degree of freedom for the analyte selection.

## Conclusions

The use of polymersomes or planar polymer membranes offer a large spectrum of possibilities for sensing devices that would be difficult to achieve using other materials and allow us to overcome barriers encounter with conventional sensors. Nanoscale control of the engineered nanomaterials remains one of the most challenging part in building nanosensor devices as the sensitivity and selectivity of such system depend not only on the recognition but also on the transduction mechanism that are directly linked to the structural conformation of the polymer material. Even though polymer sensor represents one of the most widely investigated type of sensors, polymersomes and planar polymer membrane nanosensors are still facing a development phase and tremendous effort still have to be made in order to be more widely adopted in industrial applications. One of the drawbacks that is linked with the use of nanomaterials is the identification of the potential impact on human health and on the environment. As efficient and performant as those material becomes, the development of such nanomaterials simultaneously opens the door to several unknowns that have to be considered.

## Abbreviations

ATP: adenosine triphosphate
BZ: benzoxazole
β-CD: β-cyclodextrin
CVC: current voltage characteristics
DNA: deoxyribonucleic acid
FET: field effect transistor
FRET: fluorescence resonance energy transfer
HRP: horseradish peroxidase
H_2_O_2_: hydrogen peroxide
HBPO: hydrophobic poly(3-ethyl-3-oxethanemethanol)
MT1-MMP1: matrix metalloproteinase type 1
MOF: metal organic framework
mPeg-b-Leu: methoxy-poly(ethylene glycol)-block-poly(rac-leucine)
ATP: *o*-aminothiophenol
ATP@AuNPs: *o*-aminothiophenol functionalized gold nanoparticles
OmpF: outer membrane protein F
MMMs: mixed matrix polymer membranes
PDA: polydiacetylene
PDMS: polydimethylsiloxane
PNTA: poly(*N*-[4H-1,2,4-triazol-4-yl]acrylamide)
PVC: poly(vinyl chloride)
PPy: polypyrrole
PMOXA: poly(2-methyl-2-oxazoline)
MIP: molecularly imprinted polymer
PAT: phosphinothricin acetyltransferase
PEO: poly(ethylene oxide)
pH: potential of hydrogen
RNA: ribonucleic acid
SAW: surface acoustic wave
SMZ: simazine
SO_2_: sulfur dioxide
TAA: tertiary alkanolamines
E2: 17β-estradiol
MNA: 6-mercaptonicotinic acid
